# Synthesis and crystal structure of 5,17-di­amino-11-*tert*-butyl-25,26,27,28-tetra­prop­oxy-23-[(tri­phenyl­meth­yl)amino]­calix[4]arene di­chloro­methane monosolvate

**DOI:** 10.1107/S2056989025010886

**Published:** 2026-01-01

**Authors:** Ivan Alekseev, Stanislav Bezzubov, Alexander Gorbunov, Vladimir Kovalev, Ivan Vatsouro

**Affiliations:** a Department of Chemistry, Lomonosov Moscow State University, Lenin’s Hills, 1, Moscow, 119991, Russian Federation; b N. S. Kurnakov Institute of General and Inorganic Chemistry, Russian Academy of Sciences, Leninskii pr. 31, Moscow, 119991, Russian Federation; Vienna University of Technology, Austria

**Keywords:** crystal structure, macrocycles, calix[4]arene, amines, selective modification, solvate

## Abstract

The title compound crystallizes as a di­chloro­methane monosolvate, C_63_H_73_N_3_O_4_·CH_2_Cl_2_. The main mol­ecule represents a calix[4]arene and possesses a pinched cone shape, with the two distal *para*-amino groups at the wide rim of the macrocycle being brought into close proximity due to steric repulsion between the bulky trityl-amino and *tert*-butyl groups attached to the other two distal positions of the wide rim. In the crystal, the calixarene mol­ecules are assembled by numerous C—H⋯π and van der Waals inter­actions, while the solvent mol­ecules reside in the cavities forming C—H⋯π and Cl⋯π contacts with the phenyl rings of the trityl group.

## Abstract 2

5,17-Di­amino-11-*tert*-butyl-25,26,27,28-tetra­prop­oxy-23-[(tri­phenyl­meth­yl)amino]­calix[4]arene crystallizes as a di­chloro­methane monosolvate and represents a calixarene with a pinched cone shape.

## 1. Chemical context

The synthetic availability of calixarenes and well-developed methods for their selective or exhaustive chemical modification involving phenolic oxygen atoms, aromatic *para*- and *meta*-positions to them, as well as methyl­ene bridges with control of the mol­ecular shape allow the use of calixarene macrocycles as versatile mol­ecular platforms for the development of receptor mol­ecules. The introduction of several identical or different functional groups into calixarene macrocycles leads to multifunctional derivatives capable of effectively and selectively binding cations, anions, ion pairs, and neutral mol­ecules, which can be used in the design of mol­ecular sensors or switches among others. Aromatic cavities formed by aromatic moieties of calixarenes that retain certain stereoisomeric forms (which is most important for cone-shaped calixarenes) can also participate in the binding of various ionic and neutral substrates. Thus, varying the number and nature of the functional/receptor groups surrounding the aromatic cavities of calixarenes allows the construction of receptor structures of virtually unlimited complexity and makes available, for example, host mol­ecules that are capable of efficiently and selectively binding various guest mol­ecules into host–guest complexes (Asfari *et al.*, 2001 ▸; Vicens *et al.*, 2007 ▸; Böhmer, 2003 ▸; Neri *et al.*, 2016 ▸). Grafting four or less amino groups into the *para*-positions of calix[4]arenes makes the resulting compounds attractive for further modification. So far developed synthetic routes enable the transformation of *para*-aminated calix[4]arenes into more complex (supra)­mol­ecules with valuable properties, such as urea derivatives for anion binding (Jo *et al.*, 2001 ▸; Surina *et al.*, 2024 ▸), supra­molecular containers for targeted drug delivery (Du *et al.*, 2023 ▸), bis­(calixarenes) linked to each other *via* wide rims (Lhoták, 2024 ▸), and even inherently chiral calixarenes (Tlustý *et al.*, 2022 ▸). However, in many cases, the introduction of several different substituents into the *para*-amino functions of calixarenes is a challenging task, for which two approaches can be used. The first one suggests a stepwise selective modification of the wide rim of the calix[4]arene (Timmerman *et al.*, 1994 ▸; Danila *et al.*, 2005 ▸; Bogdan *et al.*, 2004 ▸). The second route involves the selective reversible protection of *para*-aminated calix[4]arenes by introducing *tert*-butyl­oxycarbonyl (Saadioui *et al.*, 1999 ▸; Zadmard *et al.*, 2009 ▸) or trityl protecting groups (Rudzevich *et al.*, 2007 ▸).
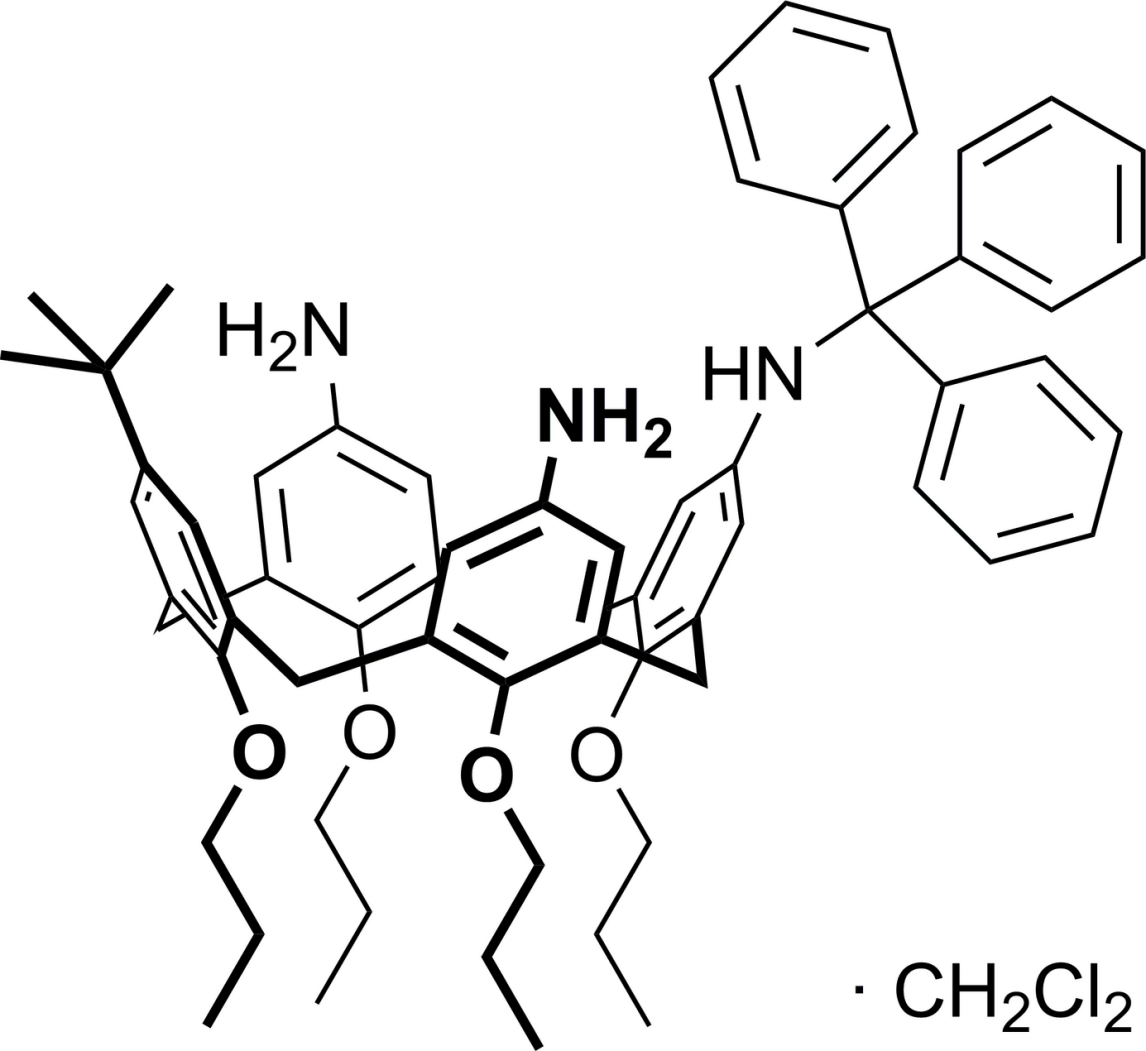


Herein, we report another successful example of selective tritylation of only one of the three amino groups at the wide rim of a calix[4]arene, which is located distally to the *tert*-butyl group and present the mol­ecular and crystal structure of the title compound, C_63_H_73_N_3_O_4_ (**1**), which crystallized as a di­chloro­methane monosolvate.

## 2. Structural commentary

**Figure 1:**
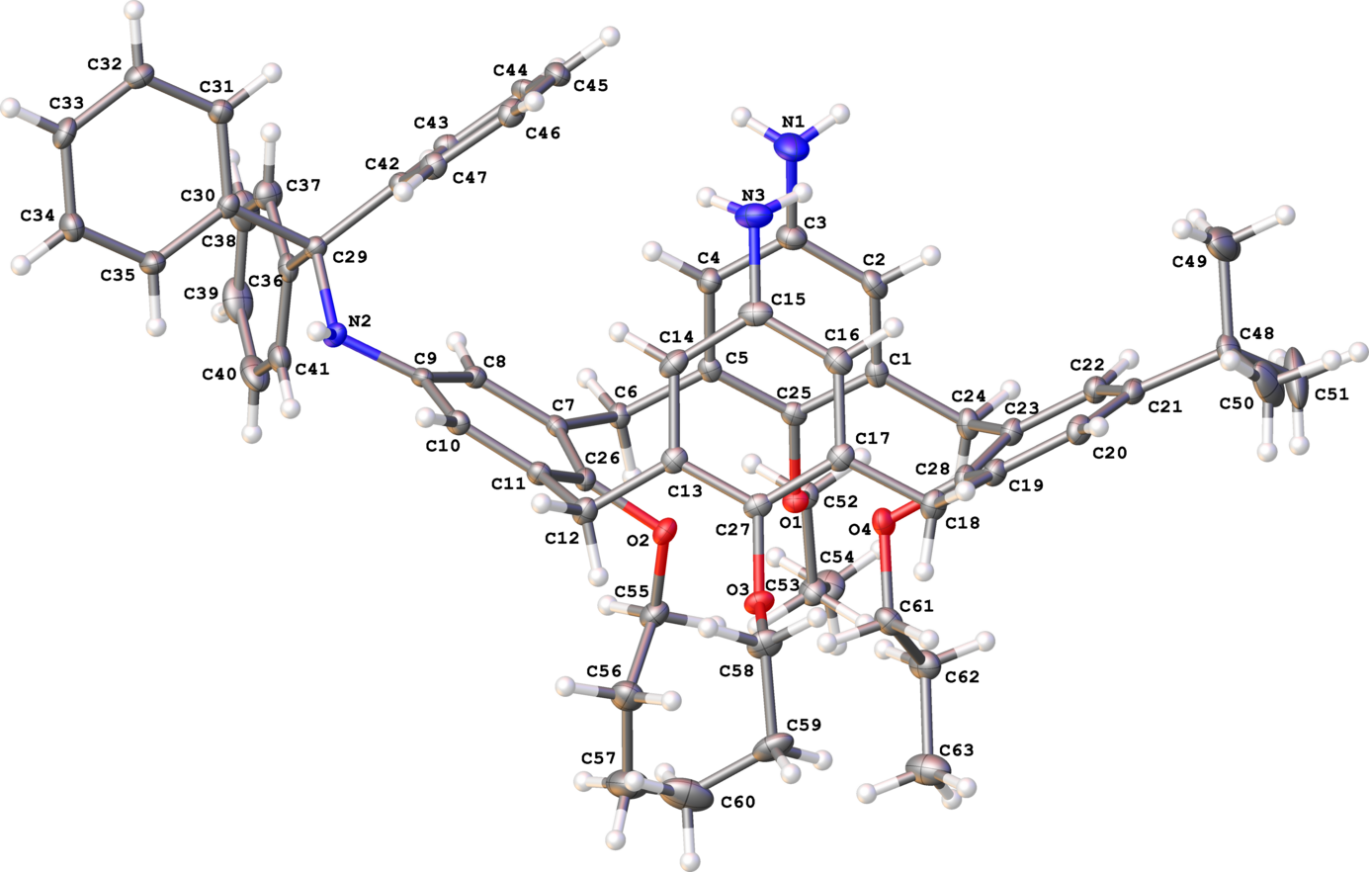
The mol­ecular structure of 5,17-di­amino-11-*tert*-butyl-25,26,27,28-tetra­prop­oxy-23-[(tri­phenyl­meth­yl)amino]­calix[4]arene (**1**), with displacement ellipsoids drawn at the 50% probability level. For clarity, the CH_2_Cl_2_ solvent mol­ecule is omitted.

The title compound occupies a general position (Fig. 1 ▸). Two bulky *para*-substituents, the tri­phenyl­methyl­amino and *tert*-butyl groups, are located in distal positions at the wide rim, which results in a pinched cone shape of the calix[4]arene. Consequently, the *para*-NH_2_-substituted phenyl rings (C1–C5/C25 and C13–C17/C27) are somewhat inclined to each other [inter­planar angle 24.72 (6)°] so that the amino-groups are brought nearer together [*d*(N1⋯N3) = 3.470 (3) Å], although no hydrogen bond is formed between these groups within the mol­ecule.

The ^1^H NMR spectrum of compound **1** is displayed in Fig. S2 in the supporting information. It comprises a set of three multiplets corresponding to the trityl group, as well as two singlets and two doublets from the aromatic protons of the calixarene. The signals corresponding to the protons from the calixarene methyl­ene bridges of the two types appear as four doublets in the middle part of the spectrum and confirm the time-averaged *C*_s_ symmetry of the mol­ecular structure, which corresponds to the calixarene substitution pattern. The spectrum also contains a singlet from the protected amino group, a broad signal from free amino groups, and three sets of signals corresponding to three different prop­yloxy groups in a 1:1:2 ratio. The ^13^C NMR spectrum is displayed in Fig. S3 in the supporting information and contains a full set of signals consistent with the mol­ecular structure, and in particular at 31.33 and 31.07 ppm there are characteristic signals reflecting the cone shape of the macrocycle, in which methyl­ene groups of two types are linked to *syn*-arranged aromatic units of the calixarene.

## 3. Supra­molecular features

**Figure 2:**
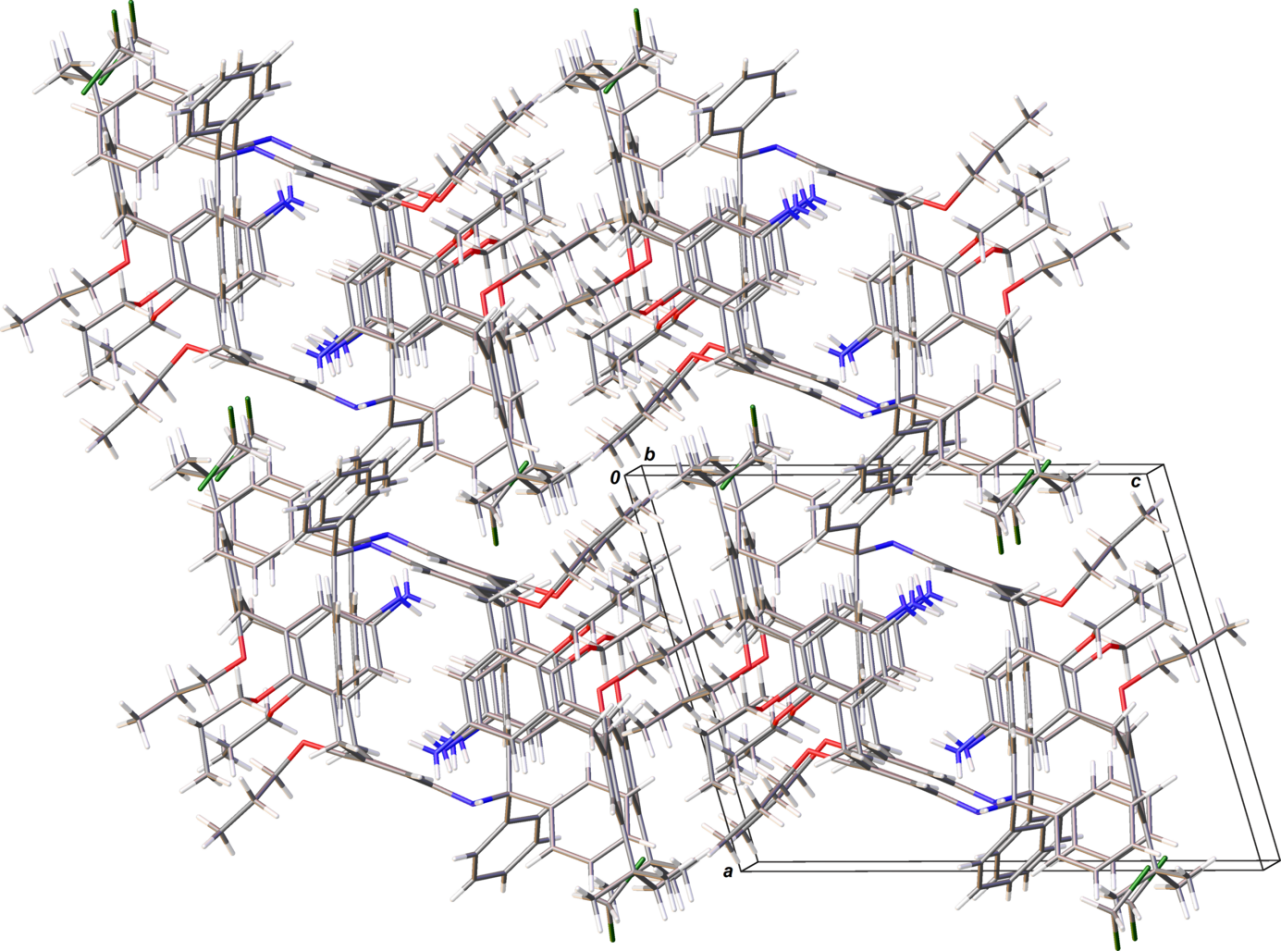
Fragment of the crystal packing of 5,17-di­amino-11-*tert*-butyl-25,26,27,28-tetra­prop­oxy-23-[(tri­phenyl­meth­yl)amino]­calix[4]arene (**1**) in a view approximately along [010], including the CH_2_Cl_2_ solvent mol­ecule.

In the crystal (Fig. 2 ▸) of the di­chloro­methane monosolvate of **1**, mol­ecules form thick layers parallel to (001) by C—H⋯π inter­actions between the H43 atom and the centroid of the C13–C17/C27 ring [2.89 (2) Å, 141 (2)°], between the H46 atom and the centroid of the C1–C5/C25 ring [2.66 (3) Å, 145 (2)°], between the H18*B* atom and the centroid of the C30–C35 ring [3.12 (2) Å, 165 (2)°], and between the H34 atom and the centroid of the C7–C11/C26 ring [2.73 (3) Å, 146 (2)°]. These layers are assembled *via* van der Waals inter­actions forming the crystal packing, in which fully ordered solvent CH_2_Cl_2_ mol­ecules (two per unit cell) reside in cavities inter­acting with the main calixarene through a C—H⋯π contact between the H64*B* atom and the centroid of the C36–C41 ring [2.68 (4) Å, 160 (3)°] and a Cl⋯π contact between the Cl2 atom and the centroid of the C30–C35 ring [3.7835 (12) Å].

## 4. Database survey

Crystal structures of similar wide-rim tetra­substituted di­amino­calix[4]arenes have not been found in the Cambridge Crystallographic Database (CSD v2025.2.0, August 2025 update; Groom *et al.*, 2016 ▸). Only two solvatomorphs of 5,11,17,23-tetra­mino-25,26,27,28-tetra­but­oxycalix[4]arene (one is a dihydrate, GAQQIB, the second is a dimethyl sulfoxide/water solvate, GAQQOH; Martins *et al.*, 2017 ▸) and one 5,17-di­amino-26,28-dimeth­yloxy-25,27-di­prop­oxycalix[4]arene (YAXPUJ; Yang *et al.*, 2005 ▸) have deposited CSD refcodes. All these calix[4]arenes possess a cone shape with the nearest NH_2_-substituents distant at *d*(N⋯N) = 6.978 (6)–8.071 (14) Å, and all of these NH_2_ groups participate in inter­molecular N—H⋯N hydrogen bonds. The reported intra­molecular N⋯N distance is more than twice as long as that in calixarene **1**, strongly suggesting that the steric bulkiness of the trityl and *tert*-butyl substituents is a key factor determining the observed proximity of the distal amino groups, likely hindering their accessibility for hydrogen bonding with neighbouring mol­ecules in the crystal.

## 5. Synthesis and crystallization

The title compound was prepared by reduction of the known cone calix[4]arene containing one *tert*-butyl and three nitro groups at the wide rim and four propyl groups at the narrow rim of the macrocycle, followed by selective tritylation of the tri­amine thus obtained (see Fig. S1 in the supporting information):

**5,17-Di­amino-11-*tert*-butyl-25,26,27,28-tetra­prop­oxy-23-[(tri­phenyl­meth­yl)amino]­calix[4]arene, 1.** To a solution of 5-*tert*-butyl-11,17,23-tri­nitro-25,26,27,28-tetra­prop­oxycalix[4]arene (1.64 g, 2.09 mmol) (Verboom *et al.*, 1992 ▸) in toluene (130 ml) a catalytic amount of ethanol-washed Raney nickel was added. The mixture was vigorously stirred under hydrogen atmosphere (∼1 bar) at room temperature for 24 h. The mixture was filtered through a paper filter and the filtrate was evaporated to dryness under reduced pressure. The residue was dissolved in di­chloro­methane (20 ml) and a solution of tri­phenyl­methyl chloride (0.73 g, 2.60 mmol) in di­chloro­methane (10 ml) was added. The reaction mixture was stirred for 2 h at room temperature, then *N*,*N*-diiso­propyl­ethyl­amine (0.45 ml, 2.60 mmol) was slowly added within 10 min and the resulting mixture was stirred for additional 12 h. Saturated aqueous NaHCO_3_ was added to the mixture, the organic layer was separated, and the aqueous layer was washed with di­chloro­methane. The combined organic phase was washed with water and brine, dried over MgSO_4_ and evaporated. The residue was purified by column chromatography (silica, hexa­ne/ethyl acetate 1:1). Single crystals suitable for X-ray analysis were grown by slow evaporation of the solvent from a solution of the compound in a di­chloro­methane/hexane mixture (1:1 *v*/*v*). Yield 0.93 g (48%), m.p. 465–467 K. ^1^H NMR spectrum (CDCl_3_, 400 MHz): δ = 7.49–7.45 (*m*, 6H; ArH_Trt_), 7.34–7.28 (*m*, 6H; ArH_Trt_), 7.24–7.19 (*m*, 3H; ArH_Trt_), 7.02 (*s*, 2H; ArH), 6.15 (*s*, 2H; ArH), 5.34 (*d*, 2H, ^4^*J*_HH_ = 2.8Hz; ArH), 4.93 (*d*, 2H, ^4^*J*_HH_ = 2.8Hz; ArH), 4.77 (*s*, 1H; ArNHC), 4.31 (*d*, 2H, ^2^*J*_HH_ = 13.4Hz; ArCH_2_Ar), 4.16 (*d*, 2H, ^2^*J*_HH_ = 13.3Hz; ArCH_2_Ar), 3.91–3.85 (*m*, 2H; OCH_2_), 3.83–3.76 (*m*, 2H; OCH_2_), 3.54–3.47 (*m*, 4H; OCH_2_), 2.96 (*d*, 2H, ^2^*J*_HH_ = 13.4Hz; ArCH_2_Ar), 2.65 (*d*, 2H, ^2^*J*_HH_ = 13.3 Hz; ArCH_2_Ar), 1.90–1.70 (*m*, 8H; OCH_2_C*H*_2_), 1.36 (*s*, 9H; C(CH_3_)_3_), 1.03 (*t*, 6H, ^3^*J*_HH_ = 7.4 Hz; CH_2_C*H*_3_), 0.81 (*t*, 3H, ^3^*J*_HH_ = 7.4 Hz; CH_2_C*H*_3_), 0.79 (*t*, 3H, ^3^*J*_HH_ = 7.4 Hz; CH_2_C*H*_3_) ppm; ^13^C NMR spectrum (100 MHz, CDCl_3_): δ = 155.83, 150.65, 148.87, 143.86, 140.39, 140.18, 136.56, 136.09, 133.80, 133.74 (C_Ar_), 129.40, 127.75, 126.51, 125.55, 117.49, 115.28, 115.10 (CH_Ar_), 76.68, 76.25, 76.10 (OCH_2_), 71.99 (C_Trt_), 34.02 (*C*(CH_3_)_3_), 31.74 (C(*C*H_3_)_3_), 31.33, 31.07 (ArCH_2_Ar), 23.42, 22.86, 22.73 (OCH_2_*C*H_2_), 10.86, 9.75, 9.69 (CH_2_*C*H_3_) ppm. ESI-MS *m*/*z*: 936.5676 [*M* + H]^+^ for C_63_H_74_N_3_O_4_ (936.5674).

## 6. Refinement

Crystal data, data collection and structure refinement details are summarized in Table 1 ▸. All hydrogen atoms were located from difference electron-density maps (Sheldrick, 2015*b* ▸) and were refined freely. The most disagreeable reflection, 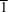
20, with an error/s.u. of more than 10 was omitted using the OMIT instruction in *SHELXL* (Sheldrick, 2015*b* ▸).

## Supplementary Material

Crystal structure: contains datablock(s) I. DOI: 10.1107/S2056989025010886/wm5780sup1.cif

Structure factors: contains datablock(s) I. DOI: 10.1107/S2056989025010886/wm5780Isup2.hkl

Supporting information file. DOI: 10.1107/S2056989025010886/wm5780Isup3.mol

Synthetic scheme and NMR spectra. DOI: 10.1107/S2056989025010886/wm5780sup4.doc

CCDC reference: 2502810

Additional supporting information:  crystallographic information; 3D view; checkCIF report

## Figures and Tables

**Table 1:** Experimental details

Crystal data	
Chemical formula	C_63_H_73_N_3_O_4_·CH_2_Cl_2_
*M*_r_	1021.17
Crystal system, space group	Triclinic, *P* 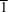
Temperature (K)	100
*a*, *b*, *c* (Å)	13.3135 (7), 14.2526 (7), 16.1475 (8)
α, β, γ (°)	84.477 (2), 73.461 (2), 73.736 (2)
*V* (Å^3^)	2819.3 (2)
*Z*	2
Radiation type	Mo *K*α
μ (mm^−1^)	0.17
Crystal size (mm)	0.14 × 0.12 × 0.09

Data collection	
Diffractometer	Bruker D8 VENTURE
Absorption correction	Multi-scan (*SADABS*; Krause *et al.*, 2015 ▸)
*T*_min_, *T*_max_	0.668, 0.746
No. of measured, independent and observed [*I* > 2σ(*I*)] reflections	55786, 15655, 13353
*R*_int_	0.041
(sin θ/λ)_max_ (Å^−1^)	0.715

Refinement	
*R*[*F*^2^ > 2σ(*F*^2^)], *wR*(*F*^2^), *S*	0.070, 0.150, 1.09
No. of reflections	15655
No. of parameters	958
H-atom treatment	All H-atom parameters refined
Δρ_max_, Δρ_min_ (e Å^−3^)	1.06, −1.15

Computer programs: *APEX3* and *SAINT* (Bruker, 2018 ▸), *SAINT* (Bruker, 2018 ▸), SHELXT (Sheldrick, 2015*a* ▸), *SHELXL* (Sheldrick, 2015*b* ▸), *OLEX2* (Dolomanov *et al.*, 2009 ▸) and *publCIF* (Westrip, 2010 ▸).

## supplementary crystallographic information

### 5,17-Diamino-11-tert-butyl-25,26,27,28-tetrapropoxy-23-[(triphenylmethyl)amino]calix[4]arene dichloromethane monosolvate Crystal data

**Table:**

C_63_H_73_N_3_O_4_·CH_2_Cl_2_	*Z* = 2
*M**_r_* = 1021.17	*F*(000) = 1092
Triclinic, *P*1	*D*_x_ = 1.203 Mg m^−^^3^
*a* = 13.3135 (7) Å	Mo *K*α radiation, λ = 0.71073 Å
*b* = 14.2526 (7) Å	Cell parameters from 9911 reflections
*c* = 16.1475 (8) Å	θ = 2.4–30.4°
α = 84.477 (2)°	µ = 0.17 mm^−^^1^
β = 73.461 (2)°	*T* = 100 K
γ = 73.736 (2)°	Block, colourless
*V* = 2819.3 (2) Å^3^	0.14 × 0.12 × 0.09 mm

### 5,17-Diamino-11-tert-butyl-25,26,27,28-tetrapropoxy-23-[(triphenylmethyl)amino]calix[4]arene dichloromethane monosolvate Data collection

**Table:**

Bruker D8 VENTURE diffractometer	15655 independent reflections
Radiation source: microfocus sealed X-ray tube	13353 reflections with *I* > 2σ(*I*)
Detector resolution: 10.4 pixels mm^-1^	*R*_int_ = 0.041
ω–scan	θ_max_ = 30.6°, θ_min_ = 2.0°
Absorption correction: multi-scan (SADABS; Krause *et al.*, 2015)	*h* = −18→19
*T*_min_ = 0.668, *T*_max_ = 0.746	*k* = −19→18
55786 measured reflections	*l* = −23→23

### 5,17-Diamino-11-tert-butyl-25,26,27,28-tetrapropoxy-23-[(triphenylmethyl)amino]calix[4]arene dichloromethane monosolvate Refinement

**Table:**

Refinement on *F*^2^	0 restraints
Least-squares matrix: full	Hydrogen site location: difference Fourier map
*R*[*F*^2^ > 2σ(*F*^2^)] = 0.070	All H-atom parameters refined
*wR*(*F*^2^) = 0.150	*w* = 1/[σ^2^(*F*_o_^2^) + (0.0421*P*)^2^ + 3.4201*P*] where *P* = (*F*_o_^2^ + 2*F*_c_^2^)/3
*S* = 1.09	(Δ/σ)_max_ < 0.001
15655 reflections	Δρ_max_ = 1.06 e Å^−^^3^
958 parameters	Δρ_min_ = −1.14 e Å^−^^3^

### 5,17-Diamino-11-tert-butyl-25,26,27,28-tetrapropoxy-23-[(triphenylmethyl)amino]calix[4]arene dichloromethane monosolvate Special details

**Table:**

Geometry. All esds (except the esd in the dihedral angle between two l.s. planes) are estimated using the full covariance matrix. The cell esds are taken into account individually in the estimation of esds in distances, angles and torsion angles; correlations between esds in cell parameters are only used when they are defined by crystal symmetry. An approximate (isotropic) treatment of cell esds is used for estimating esds involving l.s. planes.

### 5,17-Diamino-11-tert-butyl-25,26,27,28-tetrapropoxy-23-[(triphenylmethyl)amino]calix[4]arene dichloromethane monosolvate Fractional atomic coordinates and isotropic or equivalent isotropic displacement parameters (Å^2^)

**Table:**

	*x*	*y*	*z*	*U*_iso_*/*U*_eq_	
Cl1	−0.17761 (5)	0.74030 (5)	0.29380 (5)	0.04907 (19)	
Cl2	0.02596 (8)	0.77458 (9)	0.18930 (5)	0.0719 (3)	
O1	0.39809 (10)	0.57482 (8)	0.82334 (8)	0.0130 (2)	
O3	0.43194 (10)	0.97317 (9)	0.83369 (8)	0.0150 (2)	
O4	0.53467 (10)	0.72619 (9)	0.84126 (8)	0.0159 (2)	
O2	0.32400 (10)	0.80768 (9)	0.77124 (8)	0.0151 (2)	
N2	0.17016 (12)	0.91087 (11)	0.47851 (9)	0.0140 (3)	
N3	0.68766 (16)	0.83418 (13)	0.50607 (11)	0.0244 (4)	
N1	0.68916 (16)	0.59132 (15)	0.49819 (12)	0.0292 (4)	
C6	0.30856 (14)	0.65188 (12)	0.68085 (11)	0.0128 (3)	
C8	0.23661 (13)	0.78395 (12)	0.58013 (11)	0.0124 (3)	
C7	0.27472 (13)	0.75848 (12)	0.65329 (11)	0.0122 (3)	
C47	0.35973 (14)	0.69127 (12)	0.37976 (11)	0.0148 (3)	
C27	0.49694 (14)	0.94173 (12)	0.75142 (11)	0.0137 (3)	
C28	0.64526 (14)	0.69316 (13)	0.83052 (11)	0.0140 (3)	
C42	0.31754 (13)	0.79237 (12)	0.38081 (10)	0.0122 (3)	
C10	0.24559 (14)	0.94935 (12)	0.58695 (11)	0.0139 (3)	
C1	0.58202 (14)	0.55442 (12)	0.73446 (11)	0.0138 (3)	
C26	0.29009 (13)	0.83112 (12)	0.69665 (11)	0.0130 (3)	
C25	0.47019 (13)	0.58145 (12)	0.74269 (11)	0.0124 (3)	
C43	0.39043 (15)	0.85060 (13)	0.36467 (11)	0.0152 (3)	
C44	0.50150 (15)	0.80855 (13)	0.34663 (11)	0.0168 (3)	
C17	0.60657 (15)	0.89264 (12)	0.73989 (11)	0.0149 (3)	
C19	0.70775 (14)	0.75919 (13)	0.82242 (11)	0.0149 (3)	
C9	0.21748 (13)	0.88046 (12)	0.54788 (10)	0.0128 (3)	
C16	0.66908 (15)	0.85864 (13)	0.65793 (12)	0.0174 (3)	
C15	0.62350 (15)	0.86954 (13)	0.58880 (11)	0.0178 (3)	
C5	0.42999 (13)	0.61701 (11)	0.67144 (11)	0.0124 (3)	
C30	0.16112 (14)	0.91040 (12)	0.33037 (11)	0.0131 (3)	
C23	0.69412 (14)	0.59288 (13)	0.82033 (11)	0.0145 (3)	
C2	0.65296 (14)	0.55783 (13)	0.65279 (12)	0.0167 (3)	
C20	0.81861 (15)	0.72178 (14)	0.81498 (12)	0.0168 (3)	
C22	0.80504 (14)	0.55859 (13)	0.81265 (11)	0.0159 (3)	
C11	0.28220 (14)	0.92557 (12)	0.66098 (11)	0.0137 (3)	
C12	0.32764 (15)	0.99470 (13)	0.69628 (12)	0.0156 (3)	
C35	0.06024 (15)	0.98021 (14)	0.34753 (12)	0.0180 (3)	
C46	0.47162 (15)	0.64893 (13)	0.36039 (12)	0.0172 (3)	
C52	0.37646 (15)	0.48054 (12)	0.83819 (11)	0.0148 (3)	
C4	0.50305 (14)	0.62012 (13)	0.59030 (11)	0.0155 (3)	
C31	0.22480 (14)	0.89837 (13)	0.24506 (11)	0.0157 (3)	
C18	0.65780 (15)	0.86860 (13)	0.81491 (12)	0.0170 (3)	
C21	0.86874 (14)	0.62131 (13)	0.81362 (11)	0.0160 (3)	
C14	0.51361 (16)	0.91734 (13)	0.60215 (11)	0.0169 (3)	
C13	0.44907 (14)	0.95267 (12)	0.68325 (11)	0.0139 (3)	
C45	0.54271 (15)	0.70758 (13)	0.34303 (11)	0.0171 (3)	
C24	0.62712 (15)	0.52563 (13)	0.81242 (12)	0.0158 (3)	
C3	0.61489 (15)	0.58823 (13)	0.58011 (12)	0.0180 (3)	
C36	0.12153 (14)	0.77421 (12)	0.42836 (11)	0.0149 (3)	
C29	0.19477 (14)	0.84479 (12)	0.40532 (11)	0.0126 (3)	
C33	0.08945 (15)	1.02521 (14)	0.19634 (12)	0.0182 (3)	
C34	0.02543 (15)	1.03772 (14)	0.28114 (12)	0.0198 (4)	
C61	0.47345 (15)	0.76261 (13)	0.92597 (12)	0.0177 (3)	
C32	0.18882 (16)	0.95549 (14)	0.17860 (11)	0.0182 (3)	
C37	0.13198 (16)	0.70469 (14)	0.36855 (13)	0.0203 (4)	
C53	0.30390 (15)	0.47490 (13)	0.92849 (12)	0.0178 (3)	
C55	0.23960 (16)	0.79406 (14)	0.84607 (12)	0.0188 (4)	
C41	0.04084 (15)	0.77959 (14)	0.50639 (13)	0.0199 (4)	
C58	0.43971 (17)	1.06522 (14)	0.85652 (12)	0.0204 (4)	
C62	0.44563 (17)	0.68308 (14)	0.99050 (13)	0.0212 (4)	
C48	0.98923 (14)	0.58012 (14)	0.81108 (13)	0.0198 (4)	
C54	0.28347 (18)	0.37395 (15)	0.94648 (14)	0.0243 (4)	
C40	−0.02393 (16)	0.71451 (16)	0.52539 (15)	0.0266 (4)	
C59	0.3733 (2)	1.08750 (15)	0.94867 (13)	0.0264 (4)	
C38	0.06760 (17)	0.63945 (16)	0.38821 (15)	0.0271 (4)	
C56	0.18020 (18)	0.88772 (15)	0.89537 (14)	0.0256 (4)	
C63	0.3816 (2)	0.72831 (17)	1.07824 (15)	0.0329 (5)	
C39	−0.01036 (17)	0.64394 (16)	0.46688 (16)	0.0299 (5)	
C51	1.0036 (2)	0.4913 (2)	0.8724 (2)	0.0482 (8)	
C49	1.05659 (18)	0.5483 (2)	0.71925 (17)	0.0368 (6)	
C50	1.03336 (19)	0.6557 (2)	0.8400 (2)	0.0379 (6)	
C60	0.2522 (2)	1.11536 (19)	0.96144 (19)	0.0399 (6)	
C57	0.1008 (2)	0.8675 (2)	0.97914 (16)	0.0405 (6)	
C64	−0.0857 (2)	0.81192 (19)	0.2795 (2)	0.0434 (6)	
H12A	0.2931 (16)	1.0064 (15)	0.7573 (13)	0.009 (5)*	
H6A	0.2689 (16)	0.6416 (14)	0.7417 (13)	0.008 (5)*	
H24A	0.5691 (19)	0.5275 (16)	0.8636 (15)	0.019 (6)*	
H8	0.2263 (17)	0.7346 (16)	0.5516 (13)	0.012 (5)*	
H31	0.2928 (18)	0.8509 (16)	0.2330 (14)	0.013 (5)*	
H18A	0.6030 (18)	0.8936 (16)	0.8674 (14)	0.015 (5)*	
H16	0.7424 (19)	0.8244 (17)	0.6498 (14)	0.019 (6)*	
H61A	0.4091 (17)	0.8119 (15)	0.9188 (13)	0.012 (5)*	
H53A	0.3395 (18)	0.4899 (16)	0.9703 (14)	0.016 (5)*	
H45	0.6190 (19)	0.6794 (17)	0.3289 (15)	0.020 (6)*	
H6B	0.2895 (18)	0.6133 (16)	0.6443 (14)	0.019 (5)*	
H47	0.3126 (17)	0.6493 (16)	0.3937 (14)	0.014 (5)*	
H2A	0.730 (2)	0.5398 (17)	0.6462 (15)	0.023 (6)*	
H10	0.2404 (18)	1.0136 (17)	0.5614 (14)	0.019 (5)*	
H14	0.4816 (19)	0.9212 (17)	0.5568 (16)	0.024 (6)*	
H18B	0.7158 (19)	0.9006 (17)	0.8054 (15)	0.023 (6)*	
H52A	0.3413 (18)	0.4720 (17)	0.7952 (15)	0.020 (6)*	
H12B	0.3117 (18)	1.0577 (16)	0.6653 (14)	0.017 (5)*	
H4	0.4760 (19)	0.6461 (17)	0.5423 (15)	0.021 (6)*	
H37	0.184 (2)	0.7000 (17)	0.3140 (16)	0.024 (6)*	
H22	0.8384 (18)	0.4879 (17)	0.8070 (14)	0.018 (5)*	
H32	0.2333 (19)	0.9465 (17)	0.1190 (15)	0.022 (6)*	
H35	0.016 (2)	0.9902 (17)	0.4048 (16)	0.025 (6)*	
H52B	0.4465 (19)	0.4283 (17)	0.8293 (15)	0.021 (6)*	
H41	0.0297 (18)	0.8274 (17)	0.5472 (15)	0.020 (6)*	
H24B	0.6743 (19)	0.4584 (18)	0.8058 (15)	0.023 (6)*	
H43	0.3642 (19)	0.9201 (18)	0.3668 (15)	0.022 (6)*	
H58A	0.517 (2)	1.0619 (18)	0.8481 (15)	0.026 (6)*	
H44	0.5505 (19)	0.8484 (18)	0.3357 (15)	0.024 (6)*	
H46	0.4986 (19)	0.5805 (18)	0.3600 (15)	0.025 (6)*	
H61B	0.5152 (19)	0.7977 (18)	0.9482 (15)	0.024 (6)*	
H50A	1.108 (2)	0.6241 (19)	0.8427 (16)	0.031 (7)*	
H20	0.8596 (18)	0.7677 (17)	0.8097 (14)	0.020 (6)*	
H33	0.0664 (19)	1.0632 (17)	0.1506 (15)	0.021 (6)*	
H58B	0.4124 (19)	1.1166 (18)	0.8159 (15)	0.024 (6)*	
H34	−0.043 (2)	1.0843 (18)	0.2929 (16)	0.028 (6)*	
H54A	0.247 (2)	0.3592 (19)	0.9071 (17)	0.035 (7)*	
H54B	0.351 (2)	0.3231 (19)	0.9371 (17)	0.032 (7)*	
H40	−0.075 (2)	0.7178 (17)	0.5782 (16)	0.024 (6)*	
H62A	0.4020 (19)	0.6504 (17)	0.9702 (15)	0.020 (6)*	
H55A	0.2768 (19)	0.7441 (17)	0.8832 (15)	0.022 (6)*	
H2	0.1760 (18)	0.9675 (18)	0.4599 (15)	0.019 (6)*	
H56A	0.141 (2)	0.9376 (19)	0.8598 (16)	0.030 (6)*	
H1A	0.750 (2)	0.544 (2)	0.4901 (18)	0.038 (7)*	
H54C	0.243 (2)	0.3669 (19)	1.0034 (18)	0.032 (7)*	
H62B	0.511 (2)	0.6356 (18)	0.9949 (15)	0.026 (6)*	
H53B	0.236 (2)	0.5255 (18)	0.9347 (15)	0.026 (6)*	
H55B	0.1867 (19)	0.7678 (17)	0.8288 (15)	0.024 (6)*	
H3A	0.738 (2)	0.781 (2)	0.5085 (19)	0.044 (8)*	
H39	−0.055 (2)	0.599 (2)	0.4810 (18)	0.039 (7)*	
H56B	0.235 (2)	0.916 (2)	0.9076 (17)	0.035 (7)*	
H59A	0.392 (2)	1.143 (2)	0.9678 (17)	0.036 (7)*	
H1B	0.662 (2)	0.596 (2)	0.455 (2)	0.041 (8)*	
H50B	0.986 (3)	0.680 (2)	0.897 (2)	0.050 (9)*	
H38	0.077 (2)	0.593 (2)	0.3451 (17)	0.034 (7)*	
H50C	1.034 (3)	0.714 (2)	0.798 (2)	0.055 (9)*	
H57A	0.136 (3)	0.818 (2)	1.014 (2)	0.051 (9)*	
H49A	1.048 (3)	0.607 (2)	0.683 (2)	0.051 (9)*	
H51A	1.082 (3)	0.466 (2)	0.8714 (19)	0.050 (8)*	
H59B	0.393 (2)	1.031 (2)	0.9849 (18)	0.037 (7)*	
H60A	0.228 (3)	1.169 (2)	0.924 (2)	0.050 (9)*	
H3B	0.647 (2)	0.822 (2)	0.4774 (19)	0.038 (8)*	
H63A	0.364 (3)	0.681 (2)	1.120 (2)	0.052 (9)*	
H64A	−0.122 (3)	0.878 (3)	0.269 (2)	0.075 (11)*	
H60B	0.226 (3)	1.063 (2)	0.945 (2)	0.053 (9)*	
H64B	−0.051 (3)	0.801 (3)	0.331 (2)	0.073 (11)*	
H63B	0.313 (2)	0.772 (2)	1.0743 (18)	0.043 (8)*	
H63C	0.422 (2)	0.766 (2)	1.0991 (19)	0.046 (8)*	
H49B	1.136 (3)	0.521 (2)	0.7159 (19)	0.050 (8)*	
H60C	0.210 (3)	1.137 (3)	1.019 (2)	0.064 (10)*	
H49C	1.030 (3)	0.499 (3)	0.697 (2)	0.062 (10)*	
H51B	0.981 (3)	0.441 (3)	0.859 (2)	0.060 (10)*	
H57B	0.046 (3)	0.842 (2)	0.969 (2)	0.047 (8)*	
H57C	0.065 (3)	0.926 (2)	1.016 (2)	0.057 (9)*	
H51C	0.959 (3)	0.508 (3)	0.931 (3)	0.081 (13)*	

### 5,17-Diamino-11-tert-butyl-25,26,27,28-tetrapropoxy-23-[(triphenylmethyl)amino]calix[4]arene dichloromethane monosolvate Atomic displacement parameters (Å^2^)

**Table:**

	*U*^11^	*U*^22^	*U*^33^	*U*^12^	*U*^13^	*U*^23^
Cl1	0.0320 (3)	0.0282 (3)	0.0806 (5)	−0.0097 (2)	−0.0008 (3)	−0.0095 (3)
Cl2	0.0723 (6)	0.1263 (8)	0.0378 (4)	−0.0696 (6)	−0.0113 (4)	0.0193 (4)
O1	0.0150 (6)	0.0110 (5)	0.0128 (5)	−0.0048 (4)	−0.0025 (5)	0.0008 (4)
O3	0.0214 (6)	0.0118 (6)	0.0128 (6)	−0.0063 (5)	−0.0036 (5)	−0.0023 (4)
O4	0.0129 (6)	0.0163 (6)	0.0198 (6)	−0.0034 (5)	−0.0066 (5)	−0.0011 (5)
O2	0.0184 (6)	0.0163 (6)	0.0128 (6)	−0.0061 (5)	−0.0071 (5)	0.0023 (4)
N2	0.0189 (7)	0.0092 (7)	0.0128 (6)	−0.0002 (5)	−0.0061 (6)	0.0002 (5)
N3	0.0302 (9)	0.0209 (8)	0.0165 (8)	−0.0037 (7)	0.0002 (7)	−0.0026 (6)
N1	0.0212 (9)	0.0386 (11)	0.0201 (9)	−0.0042 (8)	0.0020 (7)	0.0022 (7)
C6	0.0130 (7)	0.0114 (7)	0.0151 (8)	−0.0039 (6)	−0.0051 (6)	0.0012 (6)
C8	0.0124 (7)	0.0111 (7)	0.0131 (7)	−0.0026 (6)	−0.0026 (6)	−0.0014 (6)
C7	0.0094 (7)	0.0117 (7)	0.0136 (7)	−0.0018 (6)	−0.0014 (6)	0.0002 (6)
C47	0.0174 (8)	0.0124 (8)	0.0147 (8)	−0.0057 (6)	−0.0024 (7)	−0.0015 (6)
C27	0.0198 (8)	0.0091 (7)	0.0136 (7)	−0.0070 (6)	−0.0040 (7)	0.0003 (6)
C28	0.0133 (7)	0.0163 (8)	0.0145 (7)	−0.0048 (6)	−0.0067 (6)	0.0016 (6)
C42	0.0136 (7)	0.0126 (7)	0.0104 (7)	−0.0023 (6)	−0.0042 (6)	−0.0005 (6)
C10	0.0154 (8)	0.0093 (7)	0.0151 (8)	−0.0005 (6)	−0.0038 (6)	0.0002 (6)
C1	0.0146 (8)	0.0089 (7)	0.0195 (8)	−0.0037 (6)	−0.0067 (7)	0.0000 (6)
C26	0.0114 (7)	0.0150 (8)	0.0123 (7)	−0.0027 (6)	−0.0039 (6)	0.0009 (6)
C25	0.0142 (7)	0.0085 (7)	0.0147 (7)	−0.0043 (6)	−0.0028 (6)	−0.0011 (6)
C43	0.0194 (8)	0.0109 (8)	0.0156 (8)	−0.0045 (6)	−0.0051 (7)	0.0015 (6)
C44	0.0180 (8)	0.0184 (8)	0.0165 (8)	−0.0089 (7)	−0.0055 (7)	0.0030 (6)
C17	0.0208 (8)	0.0097 (7)	0.0163 (8)	−0.0067 (6)	−0.0062 (7)	0.0021 (6)
C19	0.0175 (8)	0.0144 (8)	0.0150 (8)	−0.0058 (6)	−0.0070 (7)	0.0013 (6)
C9	0.0119 (7)	0.0136 (8)	0.0097 (7)	0.0007 (6)	−0.0015 (6)	−0.0011 (6)
C16	0.0169 (8)	0.0133 (8)	0.0206 (8)	−0.0045 (6)	−0.0030 (7)	0.0016 (6)
C15	0.0246 (9)	0.0116 (8)	0.0155 (8)	−0.0076 (7)	−0.0004 (7)	0.0010 (6)
C5	0.0138 (7)	0.0074 (7)	0.0162 (8)	−0.0027 (6)	−0.0044 (6)	−0.0005 (6)
C30	0.0158 (8)	0.0116 (7)	0.0136 (7)	−0.0041 (6)	−0.0064 (6)	0.0005 (6)
C23	0.0159 (8)	0.0147 (8)	0.0153 (8)	−0.0056 (6)	−0.0069 (7)	0.0019 (6)
C2	0.0123 (8)	0.0137 (8)	0.0238 (9)	−0.0031 (6)	−0.0041 (7)	−0.0021 (6)
C20	0.0164 (8)	0.0197 (9)	0.0179 (8)	−0.0097 (7)	−0.0068 (7)	0.0031 (6)
C22	0.0159 (8)	0.0158 (8)	0.0168 (8)	−0.0033 (6)	−0.0065 (7)	−0.0003 (6)
C11	0.0143 (7)	0.0113 (7)	0.0146 (8)	−0.0017 (6)	−0.0034 (6)	−0.0020 (6)
C12	0.0198 (8)	0.0111 (8)	0.0170 (8)	−0.0033 (6)	−0.0074 (7)	−0.0002 (6)
C35	0.0174 (8)	0.0203 (9)	0.0129 (8)	−0.0005 (7)	−0.0030 (7)	−0.0005 (6)
C46	0.0181 (8)	0.0117 (8)	0.0185 (8)	−0.0001 (6)	−0.0025 (7)	−0.0034 (6)
C52	0.0178 (8)	0.0111 (8)	0.0166 (8)	−0.0056 (6)	−0.0054 (7)	0.0022 (6)
C4	0.0167 (8)	0.0139 (8)	0.0149 (8)	−0.0030 (6)	−0.0045 (7)	0.0019 (6)
C31	0.0161 (8)	0.0151 (8)	0.0152 (8)	−0.0022 (6)	−0.0043 (7)	−0.0018 (6)
C18	0.0206 (8)	0.0137 (8)	0.0204 (9)	−0.0067 (7)	−0.0094 (7)	0.0005 (6)
C21	0.0136 (8)	0.0212 (9)	0.0140 (8)	−0.0056 (6)	−0.0047 (7)	0.0014 (6)
C14	0.0262 (9)	0.0137 (8)	0.0137 (8)	−0.0084 (7)	−0.0076 (7)	0.0025 (6)
C13	0.0187 (8)	0.0089 (7)	0.0156 (8)	−0.0060 (6)	−0.0053 (7)	0.0015 (6)
C45	0.0140 (8)	0.0191 (9)	0.0148 (8)	−0.0012 (6)	−0.0018 (7)	−0.0005 (6)
C24	0.0158 (8)	0.0117 (8)	0.0222 (9)	−0.0041 (6)	−0.0089 (7)	0.0020 (6)
C3	0.0180 (8)	0.0164 (8)	0.0172 (8)	−0.0049 (6)	−0.0008 (7)	0.0001 (6)
C36	0.0124 (7)	0.0145 (8)	0.0186 (8)	−0.0029 (6)	−0.0071 (7)	0.0035 (6)
C29	0.0141 (7)	0.0116 (7)	0.0117 (7)	−0.0024 (6)	−0.0037 (6)	−0.0004 (6)
C33	0.0223 (9)	0.0191 (9)	0.0167 (8)	−0.0068 (7)	−0.0110 (7)	0.0049 (7)
C34	0.0174 (8)	0.0189 (9)	0.0213 (9)	−0.0004 (7)	−0.0069 (7)	0.0008 (7)
C61	0.0158 (8)	0.0138 (8)	0.0220 (9)	−0.0027 (6)	−0.0035 (7)	−0.0018 (6)
C32	0.0222 (9)	0.0208 (9)	0.0123 (8)	−0.0077 (7)	−0.0039 (7)	0.0009 (6)
C37	0.0194 (9)	0.0205 (9)	0.0228 (9)	−0.0071 (7)	−0.0069 (8)	−0.0005 (7)
C53	0.0199 (9)	0.0159 (8)	0.0168 (8)	−0.0055 (7)	−0.0044 (7)	0.0038 (6)
C55	0.0239 (9)	0.0194 (9)	0.0142 (8)	−0.0069 (7)	−0.0062 (7)	0.0011 (6)
C41	0.0147 (8)	0.0211 (9)	0.0223 (9)	−0.0011 (7)	−0.0071 (7)	0.0032 (7)
C58	0.0297 (10)	0.0132 (8)	0.0200 (9)	−0.0088 (7)	−0.0056 (8)	−0.0027 (7)
C62	0.0240 (9)	0.0144 (8)	0.0221 (9)	−0.0021 (7)	−0.0046 (8)	0.0008 (7)
C48	0.0123 (8)	0.0231 (9)	0.0251 (9)	−0.0054 (7)	−0.0069 (7)	0.0017 (7)
C54	0.0280 (10)	0.0227 (10)	0.0226 (10)	−0.0130 (8)	−0.0041 (9)	0.0078 (8)
C40	0.0143 (8)	0.0321 (11)	0.0298 (11)	−0.0065 (8)	−0.0034 (8)	0.0096 (8)
C59	0.0458 (13)	0.0185 (9)	0.0161 (9)	−0.0101 (9)	−0.0074 (9)	−0.0038 (7)
C38	0.0257 (10)	0.0229 (10)	0.0394 (12)	−0.0107 (8)	−0.0155 (9)	0.0003 (9)
C56	0.0263 (10)	0.0218 (10)	0.0254 (10)	−0.0003 (8)	−0.0059 (8)	−0.0058 (8)
C63	0.0405 (13)	0.0211 (10)	0.0266 (11)	−0.0041 (9)	0.0028 (10)	0.0015 (8)
C39	0.0223 (10)	0.0276 (11)	0.0461 (13)	−0.0146 (8)	−0.0152 (10)	0.0124 (9)
C51	0.0238 (12)	0.0535 (17)	0.072 (2)	−0.0148 (11)	−0.0284 (14)	0.0359 (15)
C49	0.0172 (10)	0.0556 (16)	0.0359 (13)	−0.0055 (10)	−0.0047 (9)	−0.0120 (12)
C50	0.0188 (10)	0.0399 (14)	0.0611 (17)	−0.0039 (9)	−0.0189 (11)	−0.0171 (12)
C60	0.0415 (14)	0.0282 (12)	0.0436 (14)	−0.0128 (10)	0.0076 (12)	−0.0178 (11)
C57	0.0392 (14)	0.0439 (15)	0.0248 (11)	0.0029 (12)	0.0003 (11)	−0.0057 (10)
C64	0.0327 (12)	0.0261 (12)	0.076 (2)	−0.0076 (10)	−0.0243 (13)	0.0066 (12)

### 5,17-Diamino-11-tert-butyl-25,26,27,28-tetrapropoxy-23-[(triphenylmethyl)amino]calix[4]arene dichloromethane monosolvate Geometric parameters (Å, º)

**Table:**

Cl1—C64	1.757 (3)	C52—H52B	1.00 (2)
Cl2—C64	1.758 (3)	C4—C3	1.395 (3)
O1—C25	1.393 (2)	C4—H4	0.95 (2)
O1—C52	1.436 (2)	C31—C32	1.396 (2)
O3—C27	1.399 (2)	C31—H31	0.95 (2)
O3—C58	1.435 (2)	C18—H18A	0.97 (2)
O4—C28	1.379 (2)	C18—H18B	0.97 (2)
O4—C61	1.439 (2)	C21—C48	1.536 (2)
O2—C26	1.383 (2)	C14—C13	1.397 (2)
O2—C55	1.437 (2)	C14—H14	0.94 (2)
N2—C9	1.412 (2)	C45—H45	0.95 (2)
N2—C29	1.487 (2)	C24—H24A	0.95 (2)
N2—H2	0.85 (2)	C24—H24B	0.98 (2)
N3—C15	1.417 (2)	C36—C29	1.540 (2)
N3—H3A	0.86 (3)	C36—C37	1.402 (3)
N3—H3B	0.86 (3)	C36—C41	1.396 (3)
N1—C3	1.414 (2)	C33—C34	1.389 (3)
N1—H1A	0.88 (3)	C33—C32	1.387 (3)
N1—H1B	0.86 (3)	C33—H33	0.94 (2)
C6—C7	1.520 (2)	C34—H34	0.94 (3)
C6—C5	1.519 (2)	C61—C62	1.515 (3)
C6—H6A	0.99 (2)	C61—H61A	0.97 (2)
C6—H6B	0.97 (2)	C61—H61B	1.00 (2)
C8—C7	1.393 (2)	C32—H32	0.98 (2)
C8—C9	1.403 (2)	C37—C38	1.391 (3)
C8—H8	0.94 (2)	C37—H37	0.95 (2)
C7—C26	1.394 (2)	C53—C54	1.524 (3)
C47—C42	1.392 (2)	C53—H53A	1.00 (2)
C47—C46	1.396 (2)	C53—H53B	0.97 (2)
C47—H47	0.95 (2)	C55—C56	1.515 (3)
C27—C17	1.397 (2)	C55—H55A	1.00 (2)
C27—C13	1.397 (2)	C55—H55B	1.00 (2)
C28—C19	1.396 (2)	C41—C40	1.393 (3)
C28—C23	1.399 (2)	C41—H41	0.95 (2)
C42—C43	1.401 (2)	C58—C59	1.511 (3)
C42—C29	1.546 (2)	C58—H58A	0.98 (2)
C10—C9	1.401 (2)	C58—H58B	1.00 (2)
C10—C11	1.394 (2)	C62—C63	1.527 (3)
C10—H10	0.96 (2)	C62—H62A	0.97 (2)
C1—C25	1.400 (2)	C62—H62B	0.96 (3)
C1—C2	1.392 (3)	C48—C51	1.533 (3)
C1—C24	1.519 (2)	C48—C49	1.534 (3)
C26—C11	1.400 (2)	C48—C50	1.527 (3)
C25—C5	1.398 (2)	C54—H54A	0.97 (3)
C43—C44	1.387 (3)	C54—H54B	0.97 (3)
C43—H43	0.95 (2)	C54—H54C	0.93 (3)
C44—C45	1.390 (3)	C40—C39	1.386 (3)
C44—H44	0.95 (2)	C40—H40	0.92 (2)
C17—C16	1.396 (3)	C59—C60	1.505 (4)
C17—C18	1.520 (2)	C59—H59A	1.00 (3)
C19—C20	1.396 (2)	C59—H59B	0.97 (3)
C19—C18	1.522 (2)	C38—C39	1.387 (3)
C16—C15	1.392 (3)	C38—H38	0.97 (3)
C16—H16	0.94 (2)	C56—C57	1.520 (3)
C15—C14	1.393 (3)	C56—H56A	0.99 (3)
C5—C4	1.397 (2)	C56—H56B	1.00 (3)
C30—C35	1.401 (2)	C63—H63A	0.93 (3)
C30—C31	1.395 (2)	C63—H63B	0.97 (3)
C30—C29	1.548 (2)	C63—H63C	1.00 (3)
C23—C22	1.392 (2)	C39—H39	0.97 (3)
C23—C24	1.516 (2)	C51—H51A	1.00 (3)
C2—C3	1.393 (3)	C51—H51B	0.92 (4)
C2—H2A	0.96 (2)	C51—H51C	0.97 (4)
C20—C21	1.400 (3)	C49—H49A	0.97 (3)
C20—H20	0.95 (2)	C49—H49B	1.01 (3)
C22—C21	1.398 (2)	C49—H49C	1.00 (4)
C22—H22	0.98 (2)	C50—H50A	0.98 (3)
C11—C12	1.517 (2)	C50—H50B	0.99 (3)
C12—C13	1.518 (2)	C50—H50C	1.02 (3)
C12—H12A	0.97 (2)	C60—H60A	0.97 (3)
C12—H12B	0.98 (2)	C60—H60B	0.99 (3)
C35—C34	1.392 (3)	C60—H60C	0.97 (4)
C35—H35	0.94 (2)	C57—H57A	0.96 (3)
C46—C45	1.386 (3)	C57—H57B	0.97 (3)
C46—H46	0.94 (2)	C57—H57C	0.99 (3)
C52—C53	1.512 (2)	C64—H64A	0.96 (4)
C52—H52A	0.97 (2)	C64—H64B	1.04 (4)
			
C25—O1—C52	111.75 (12)	C23—C24—H24A	110.3 (14)
C27—O3—C58	113.95 (13)	C23—C24—H24B	108.3 (14)
C28—O4—C61	114.33 (13)	H24A—C24—H24B	108.1 (19)
C26—O2—C55	113.80 (13)	C2—C3—N1	120.01 (17)
C9—N2—C29	120.77 (13)	C2—C3—C4	119.02 (16)
C9—N2—H2	111.2 (16)	C4—C3—N1	120.89 (17)
C29—N2—H2	110.6 (15)	C37—C36—C29	119.83 (16)
C15—N3—H3A	113 (2)	C41—C36—C29	121.96 (16)
C15—N3—H3B	110.1 (19)	C41—C36—C37	118.15 (17)
H3A—N3—H3B	108 (3)	N2—C29—C42	108.81 (13)
C3—N1—H1A	114.3 (18)	N2—C29—C30	106.35 (13)
C3—N1—H1B	115 (2)	N2—C29—C36	110.35 (14)
H1A—N1—H1B	111 (3)	C42—C29—C30	111.87 (13)
C7—C6—H6A	110.9 (11)	C36—C29—C42	113.55 (13)
C7—C6—H6B	107.8 (13)	C36—C29—C30	105.67 (13)
C5—C6—C7	111.64 (13)	C34—C33—H33	121.2 (14)
C5—C6—H6A	109.1 (12)	C32—C33—C34	119.36 (16)
C5—C6—H6B	109.3 (13)	C32—C33—H33	119.5 (14)
H6A—C6—H6B	108.1 (17)	C35—C34—H34	120.4 (15)
C7—C8—C9	121.07 (15)	C33—C34—C35	120.33 (17)
C7—C8—H8	118.4 (13)	C33—C34—H34	119.2 (15)
C9—C8—H8	120.5 (13)	O4—C61—C62	113.25 (15)
C8—C7—C6	120.41 (15)	O4—C61—H61A	106.6 (12)
C8—C7—C26	119.10 (15)	O4—C61—H61B	109.6 (14)
C26—C7—C6	120.27 (15)	C62—C61—H61A	112.3 (12)
C42—C47—C46	121.06 (16)	C62—C61—H61B	108.9 (14)
C42—C47—H47	120.5 (13)	H61A—C61—H61B	105.8 (18)
C46—C47—H47	118.4 (13)	C31—C32—H32	120.2 (14)
C17—C27—O3	119.78 (15)	C33—C32—C31	120.58 (17)
C17—C27—C13	120.73 (16)	C33—C32—H32	119.2 (14)
C13—C27—O3	119.27 (15)	C36—C37—H37	121.1 (15)
O4—C28—C19	120.51 (15)	C38—C37—C36	120.91 (19)
O4—C28—C23	118.30 (15)	C38—C37—H37	118.0 (15)
C19—C28—C23	120.95 (16)	C52—C53—C54	110.34 (16)
C47—C42—C43	118.10 (15)	C52—C53—H53A	108.5 (13)
C47—C42—C29	124.18 (15)	C52—C53—H53B	109.3 (14)
C43—C42—C29	117.56 (14)	C54—C53—H53A	111.2 (13)
C9—C10—H10	118.9 (13)	C54—C53—H53B	110.9 (14)
C11—C10—C9	121.15 (15)	H53A—C53—H53B	106.5 (19)
C11—C10—H10	119.9 (14)	O2—C55—C56	112.36 (16)
C25—C1—C24	121.74 (15)	O2—C55—H55A	105.6 (13)
C2—C1—C25	118.67 (16)	O2—C55—H55B	110.1 (14)
C2—C1—C24	119.54 (15)	C56—C55—H55A	110.2 (13)
O2—C26—C7	119.82 (15)	C56—C55—H55B	109.6 (14)
O2—C26—C11	119.24 (15)	H55A—C55—H55B	108.9 (19)
C7—C26—C11	120.63 (15)	C36—C41—H41	120.3 (14)
O1—C25—C1	119.51 (15)	C40—C41—C36	120.57 (19)
O1—C25—C5	119.69 (15)	C40—C41—H41	119.1 (14)
C5—C25—C1	120.79 (15)	O3—C58—C59	109.06 (15)
C42—C43—H43	120.4 (14)	O3—C58—H58A	108.8 (14)
C44—C43—C42	120.83 (16)	O3—C58—H58B	108.6 (14)
C44—C43—H43	118.8 (14)	C59—C58—H58A	112.3 (14)
C43—C44—C45	120.45 (16)	C59—C58—H58B	111.0 (14)
C43—C44—H44	120.4 (14)	H58A—C58—H58B	107 (2)
C45—C44—H44	119.1 (15)	C61—C62—C63	109.53 (16)
C27—C17—C18	122.07 (16)	C61—C62—H62A	109.2 (14)
C16—C17—C27	118.89 (16)	C61—C62—H62B	109.7 (15)
C16—C17—C18	118.88 (16)	C63—C62—H62A	109.7 (14)
C28—C19—C18	120.67 (15)	C63—C62—H62B	110.5 (15)
C20—C19—C28	118.15 (16)	H62A—C62—H62B	108 (2)
C20—C19—C18	121.01 (15)	C51—C48—C21	110.08 (16)
C8—C9—N2	122.71 (15)	C51—C48—C49	108.7 (2)
C10—C9—N2	118.93 (15)	C49—C48—C21	110.17 (16)
C10—C9—C8	118.36 (15)	C50—C48—C21	111.36 (16)
C17—C16—H16	119.6 (14)	C50—C48—C51	107.4 (2)
C15—C16—C17	121.38 (17)	C50—C48—C49	108.98 (19)
C15—C16—H16	118.9 (14)	C53—C54—H54A	111.9 (16)
C16—C15—N3	120.83 (18)	C53—C54—H54B	111.0 (16)
C16—C15—C14	118.68 (16)	C53—C54—H54C	112.0 (16)
C14—C15—N3	120.48 (17)	H54A—C54—H54B	105 (2)
C25—C5—C6	121.11 (15)	H54A—C54—H54C	109 (2)
C4—C5—C6	119.68 (15)	H54B—C54—H54C	107 (2)
C4—C5—C25	119.21 (15)	C41—C40—H40	119.1 (15)
C35—C30—C29	119.16 (15)	C39—C40—C41	120.7 (2)
C31—C30—C35	118.53 (16)	C39—C40—H40	120.2 (15)
C31—C30—C29	122.19 (15)	C58—C59—H59A	108.0 (16)
C28—C23—C24	119.49 (15)	C58—C59—H59B	108.6 (16)
C22—C23—C28	118.59 (16)	C60—C59—C58	114.57 (19)
C22—C23—C24	121.81 (16)	C60—C59—H59A	107.9 (15)
C1—C2—C3	121.46 (16)	C60—C59—H59B	109.3 (16)
C1—C2—H2A	119.6 (14)	H59A—C59—H59B	108 (2)
C3—C2—H2A	118.9 (14)	C37—C38—H38	118.7 (16)
C19—C20—C21	122.35 (16)	C39—C38—C37	120.3 (2)
C19—C20—H20	116.9 (14)	C39—C38—H38	120.9 (16)
C21—C20—H20	120.7 (14)	C55—C56—C57	110.45 (19)
C23—C22—C21	122.05 (16)	C55—C56—H56A	111.1 (15)
C23—C22—H22	118.1 (13)	C55—C56—H56B	108.5 (16)
C21—C22—H22	119.9 (13)	C57—C56—H56A	110.1 (15)
C10—C11—C26	118.93 (15)	C57—C56—H56B	110.4 (15)
C10—C11—C12	121.33 (15)	H56A—C56—H56B	106 (2)
C26—C11—C12	119.22 (15)	C62—C63—H63A	112.3 (19)
C11—C12—C13	109.95 (14)	C62—C63—H63B	110.0 (17)
C11—C12—H12A	111.8 (12)	C62—C63—H63C	111.6 (17)
C11—C12—H12B	107.4 (13)	H63A—C63—H63B	106 (3)
C13—C12—H12A	108.9 (12)	H63A—C63—H63C	108 (3)
C13—C12—H12B	111.2 (13)	H63B—C63—H63C	108 (2)
H12A—C12—H12B	107.5 (17)	C40—C39—C38	119.31 (19)
C30—C35—H35	120.2 (15)	C40—C39—H39	119.9 (17)
C34—C35—C30	120.71 (17)	C38—C39—H39	120.8 (17)
C34—C35—H35	119.1 (15)	C48—C51—H51A	109.6 (18)
C47—C46—H46	119.8 (15)	C48—C51—H51B	114 (2)
C45—C46—C47	120.09 (16)	C48—C51—H51C	111 (2)
C45—C46—H46	120.1 (15)	H51A—C51—H51B	108 (3)
O1—C52—C53	109.65 (14)	H51A—C51—H51C	111 (3)
O1—C52—H52A	107.9 (13)	H51B—C51—H51C	104 (3)
O1—C52—H52B	109.7 (13)	C48—C49—H49A	106.0 (19)
C53—C52—H52A	110.8 (13)	C48—C49—H49B	111.6 (17)
C53—C52—H52B	111.2 (13)	C48—C49—H49C	111.7 (19)
H52A—C52—H52B	107.6 (19)	H49A—C49—H49B	108 (3)
C5—C4—H4	119.3 (14)	H49A—C49—H49C	109 (3)
C3—C4—C5	120.66 (16)	H49B—C49—H49C	110 (3)
C3—C4—H4	120.0 (14)	C48—C50—H50A	108.3 (15)
C30—C31—C32	120.47 (16)	C48—C50—H50B	108.9 (18)
C30—C31—H31	118.9 (13)	C48—C50—H50C	111.3 (18)
C32—C31—H31	120.6 (13)	H50A—C50—H50B	111 (2)
C17—C18—C19	111.29 (14)	H50A—C50—H50C	109 (2)
C17—C18—H18A	108.7 (13)	H50B—C50—H50C	108 (3)
C17—C18—H18B	109.4 (14)	C59—C60—H60A	112.7 (19)
C19—C18—H18A	111.5 (13)	C59—C60—H60B	113.0 (18)
C19—C18—H18B	107.4 (14)	C59—C60—H60C	114 (2)
H18A—C18—H18B	108.4 (19)	H60A—C60—H60B	102 (3)
C20—C21—C48	122.28 (16)	H60A—C60—H60C	104 (3)
C22—C21—C20	117.19 (16)	H60B—C60—H60C	110 (3)
C22—C21—C48	120.52 (16)	C56—C57—H57A	111.5 (19)
C15—C14—C13	121.29 (16)	C56—C57—H57B	111.7 (18)
C15—C14—H14	118.9 (15)	C56—C57—H57C	113.0 (19)
C13—C14—H14	119.7 (15)	H57A—C57—H57B	105 (3)
C27—C13—C12	121.95 (15)	H57A—C57—H57C	106 (3)
C14—C13—C27	118.95 (16)	H57B—C57—H57C	109 (3)
C14—C13—C12	118.93 (15)	Cl1—C64—Cl2	111.21 (17)
C44—C45—H45	120.0 (14)	Cl1—C64—H64A	108 (2)
C46—C45—C44	119.41 (16)	Cl1—C64—H64B	110 (2)
C46—C45—H45	120.6 (14)	Cl2—C64—H64A	107 (2)
C1—C24—H24A	110.0 (14)	Cl2—C64—H64B	104 (2)
C1—C24—H24B	109.4 (14)	H64A—C64—H64B	115 (3)
C23—C24—C1	110.72 (14)		
			
O1—C25—C5—C6	2.0 (2)	C15—C14—C13—C27	1.6 (3)
O1—C25—C5—C4	−177.77 (15)	C15—C14—C13—C12	−173.74 (16)
O1—C52—C53—C54	−177.95 (15)	C5—C6—C7—C8	112.96 (17)
O3—C27—C17—C16	177.62 (15)	C5—C6—C7—C26	−61.6 (2)
O3—C27—C17—C18	2.2 (2)	C5—C4—C3—N1	−179.94 (17)
O3—C27—C13—C12	−2.1 (2)	C5—C4—C3—C2	−3.3 (3)
O3—C27—C13—C14	−177.29 (15)	C30—C35—C34—C33	1.4 (3)
O3—C58—C59—C60	−72.2 (2)	C30—C31—C32—C33	0.2 (3)
O4—C28—C19—C20	177.61 (15)	C23—C28—C19—C20	−8.1 (3)
O4—C28—C19—C18	−7.0 (2)	C23—C28—C19—C18	167.23 (16)
O4—C28—C23—C22	−177.67 (15)	C23—C22—C21—C20	−5.5 (3)
O4—C28—C23—C24	6.1 (2)	C23—C22—C21—C48	175.90 (16)
O4—C61—C62—C63	179.66 (17)	C2—C1—C25—O1	177.48 (15)
O2—C26—C11—C10	−178.54 (15)	C2—C1—C25—C5	−4.0 (2)
O2—C26—C11—C12	9.6 (2)	C2—C1—C24—C23	57.1 (2)
O2—C55—C56—C57	−173.55 (18)	C20—C19—C18—C17	119.38 (18)
N3—C15—C14—C13	179.98 (16)	C20—C21—C48—C51	137.9 (2)
C6—C7—C26—O2	−7.8 (2)	C20—C21—C48—C49	−102.2 (2)
C6—C7—C26—C11	165.76 (15)	C20—C21—C48—C50	18.9 (3)
C6—C5—C4—C3	−179.83 (16)	C22—C23—C24—C1	−116.08 (18)
C8—C7—C26—O2	177.59 (15)	C22—C21—C48—C51	−43.6 (3)
C8—C7—C26—C11	−8.9 (2)	C22—C21—C48—C49	76.4 (2)
C7—C6—C5—C25	120.27 (17)	C22—C21—C48—C50	−162.6 (2)
C7—C6—C5—C4	−59.9 (2)	C11—C10—C9—N2	173.43 (15)
C7—C8—C9—N2	−174.41 (15)	C11—C10—C9—C8	−5.9 (2)
C7—C8—C9—C10	4.9 (2)	C11—C12—C13—C27	−126.90 (17)
C7—C26—C11—C10	7.9 (2)	C11—C12—C13—C14	48.3 (2)
C7—C26—C11—C12	−163.94 (15)	C35—C30—C31—C32	0.6 (3)
C47—C42—C43—C44	1.2 (2)	C35—C30—C29—N2	−46.3 (2)
C47—C42—C29—N2	122.89 (17)	C35—C30—C29—C42	−165.01 (15)
C47—C42—C29—C30	−119.91 (17)	C35—C30—C29—C36	70.96 (19)
C47—C42—C29—C36	−0.4 (2)	C46—C47—C42—C43	−2.1 (3)
C47—C46—C45—C44	1.2 (3)	C46—C47—C42—C29	−177.33 (16)
C27—O3—C58—C59	−175.61 (16)	C52—O1—C25—C1	−89.46 (18)
C27—C17—C16—C15	−2.4 (3)	C52—O1—C25—C5	91.97 (17)
C27—C17—C18—C19	122.24 (18)	C31—C30—C35—C34	−1.4 (3)
C28—O4—C61—C62	−84.89 (19)	C31—C30—C29—N2	137.79 (16)
C28—C19—C20—C21	1.3 (3)	C31—C30—C29—C42	19.1 (2)
C28—C19—C18—C17	−55.8 (2)	C31—C30—C29—C36	−104.92 (18)
C28—C23—C22—C21	−0.9 (3)	C18—C17—C16—C15	173.21 (16)
C28—C23—C24—C1	60.1 (2)	C18—C19—C20—C21	−173.98 (16)
C42—C47—C46—C45	1.0 (3)	C13—C27—C17—C16	3.0 (2)
C42—C43—C44—C45	0.9 (3)	C13—C27—C17—C18	−172.40 (15)
C10—C11—C12—C13	−110.23 (18)	C24—C1—C25—O1	−5.0 (2)
C1—C25—C5—C6	−176.50 (15)	C24—C1—C25—C5	173.55 (15)
C1—C25—C5—C4	3.7 (2)	C24—C1—C2—C3	−176.97 (16)
C1—C2—C3—N1	179.67 (17)	C24—C23—C22—C21	175.26 (16)
C1—C2—C3—C4	3.0 (3)	C36—C37—C38—C39	1.7 (3)
C26—O2—C55—C56	−91.20 (18)	C36—C41—C40—C39	−0.7 (3)
C26—C11—C12—C13	61.4 (2)	C29—N2—C9—C8	−36.1 (2)
C25—O1—C52—C53	176.33 (14)	C29—N2—C9—C10	144.59 (16)
C25—C1—C2—C3	0.6 (3)	C29—C42—C43—C44	176.72 (15)
C25—C1—C24—C23	−120.40 (17)	C29—C30—C35—C34	−177.38 (17)
C25—C5—C4—C3	0.0 (2)	C29—C30—C31—C32	176.47 (16)
C43—C42—C29—N2	−52.37 (19)	C29—C36—C37—C38	179.29 (17)
C43—C42—C29—C30	64.82 (19)	C29—C36—C41—C40	−179.86 (17)
C43—C42—C29—C36	−175.68 (15)	C34—C33—C32—C31	−0.2 (3)
C43—C44—C45—C46	−2.1 (3)	C61—O4—C28—C19	−74.2 (2)
C17—C27—C13—C12	172.56 (15)	C61—O4—C28—C23	111.37 (17)
C17—C27—C13—C14	−2.7 (2)	C32—C33—C34—C35	−0.6 (3)
C17—C16—C15—N3	−179.61 (17)	C37—C36—C29—N2	−179.40 (15)
C17—C16—C15—C14	1.4 (3)	C37—C36—C29—C42	−56.9 (2)
C19—C28—C23—C22	7.9 (3)	C37—C36—C29—C30	66.03 (19)
C19—C28—C23—C24	−168.34 (16)	C37—C36—C41—C40	2.8 (3)
C19—C20—C21—C22	5.3 (3)	C37—C38—C39—C40	0.5 (3)
C19—C20—C21—C48	−176.13 (17)	C55—O2—C26—C7	−76.54 (19)
C9—N2—C29—C42	−41.7 (2)	C55—O2—C26—C11	109.84 (17)
C9—N2—C29—C30	−162.35 (15)	C41—C36—C29—N2	3.3 (2)
C9—N2—C29—C36	83.51 (18)	C41—C36—C29—C42	125.78 (17)
C9—C8—C7—C6	−172.25 (15)	C41—C36—C29—C30	−111.25 (17)
C9—C8—C7—C26	2.4 (2)	C41—C36—C37—C38	−3.3 (3)
C9—C10—C11—C26	−0.4 (3)	C41—C40—C39—C38	−1.0 (3)
C9—C10—C11—C12	171.28 (16)	C58—O3—C27—C17	83.36 (19)
C16—C17—C18—C19	−53.2 (2)	C58—O3—C27—C13	−101.97 (18)
C16—C15—C14—C13	−1.0 (3)		
