# Supplementary material for: Synthesis and crystal structure of 5,17-di­amino-11-tert-butyl-25,26,27,28-tetra­prop­oxy-23-[(tri­phenyl­meth­yl)amino]­calix[4]arene di­chloro­methane monosolvate
Source: Acta Crystallogr E Crystallogr Commun. 2026 Jan 1;82(Pt 1):24–7. doi: 10.1107/S2056989025010886 (PMC12810279; doi:10.1107/S2056989025010886)
Supplement: Supplementary file 4 [file e-82-00024-sup4.doc]

Synthesis and crystal structure of 5,17-diamino-11-*tert*-butyl-25,26,27,28-tetrapropoxy-23-[(triphenylmethyl)amino]calix[4]arene dichloromethane monosolvate

## Ivan Alekseev, Stanislav Bezzubov, Alexander Gorbunov, Vladimir Kovalev and Ivan Vatsouro

**Supporting Information**


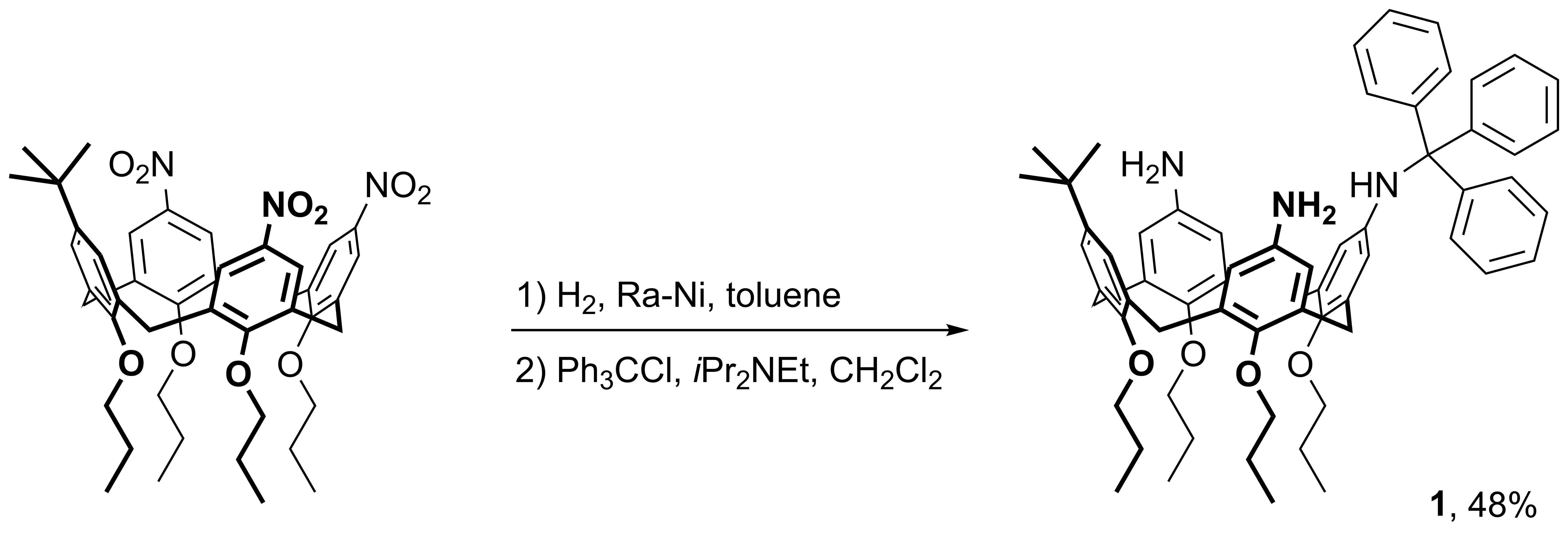


**Figure S1.** The scheme of the synthesis of compound **1**.


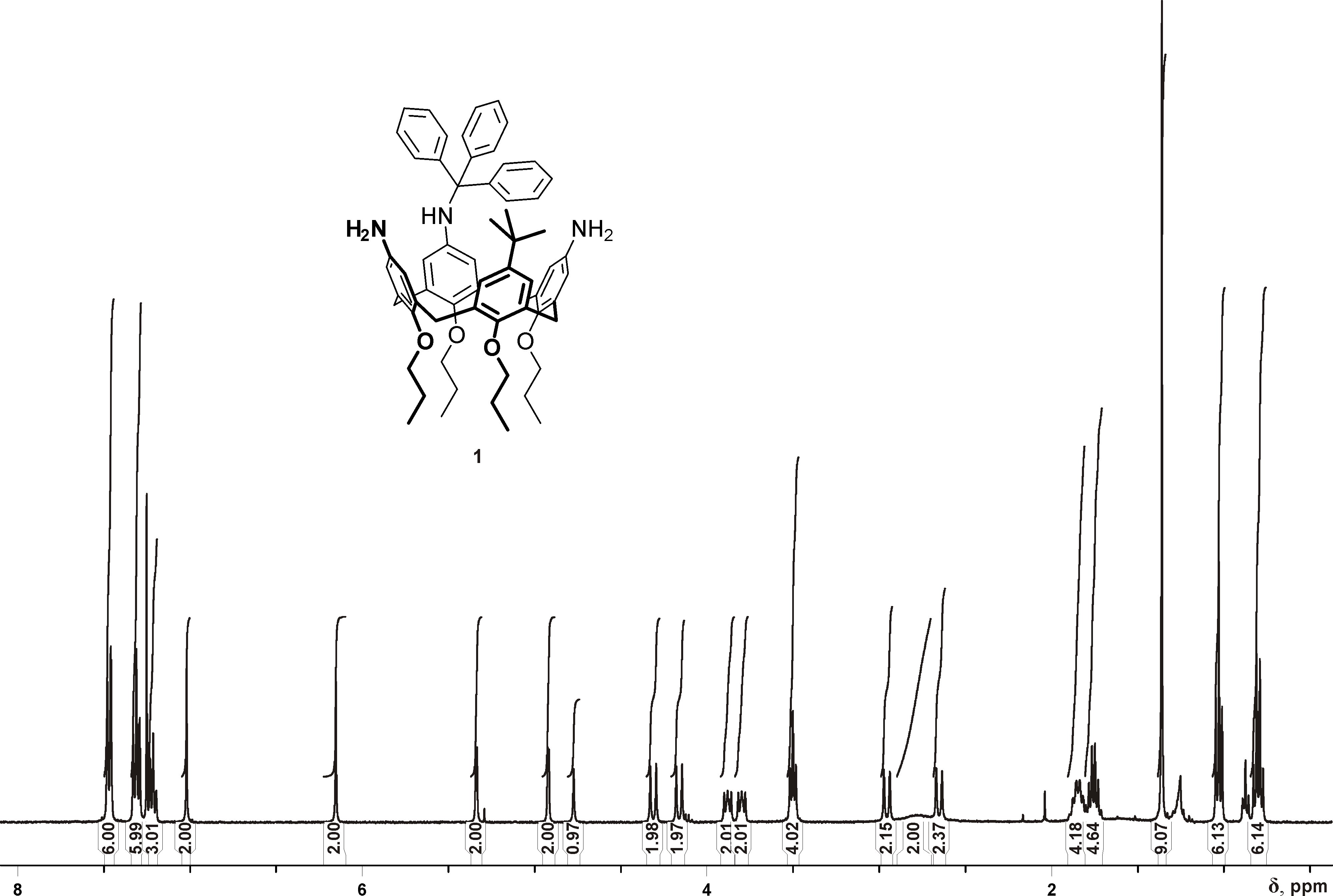


**Figure S2.** ^1^H NMR spectrum of compound **1** (400 MHz, CDCl_3_).


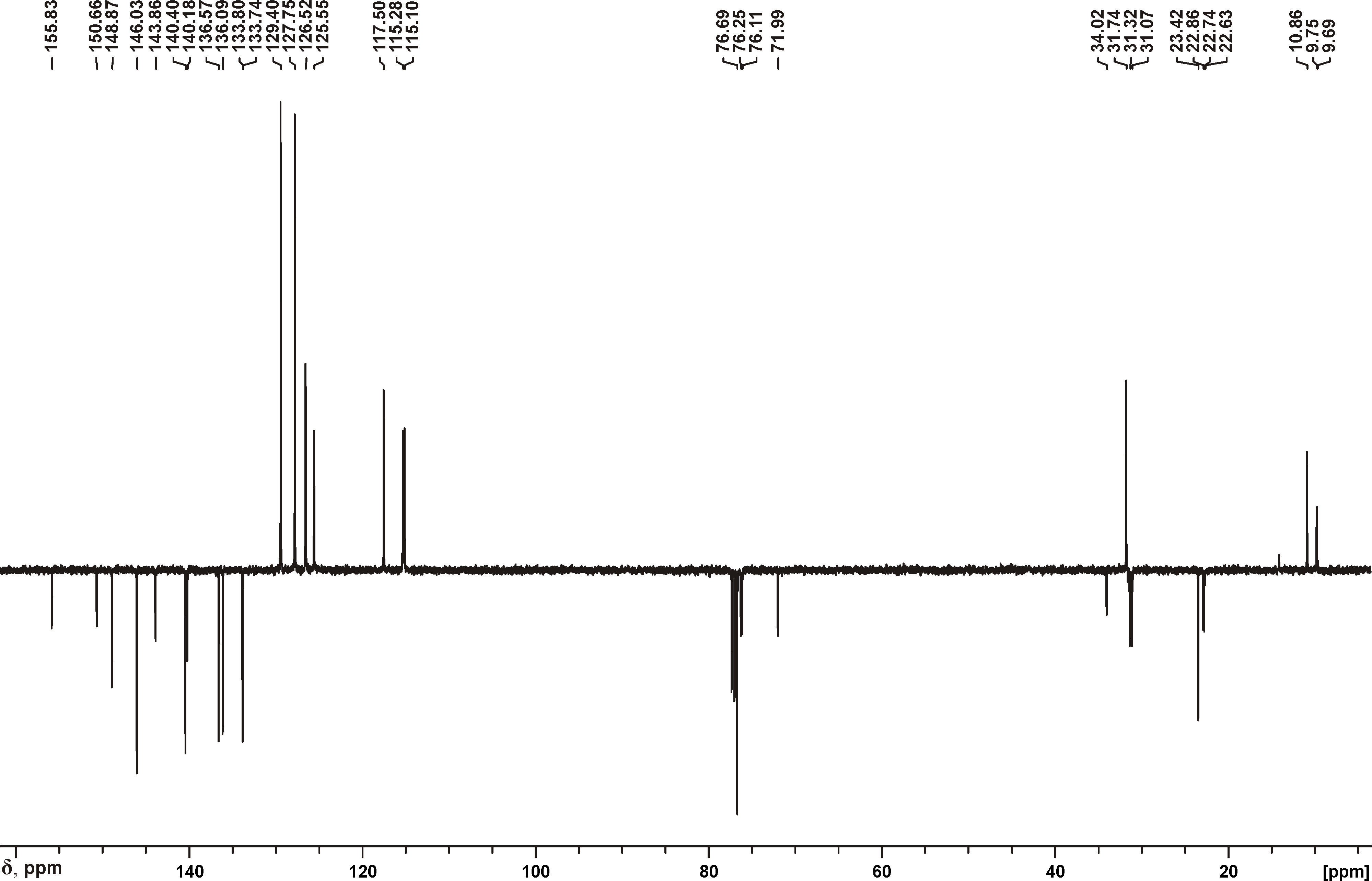


**Figure S3.** ^13^C NMR spectrum (APT) of compound **1** (100 MHz, CDCl_3_).
